# Early Bacterial Colonization and Antibiotic Resistance Gene Acquisition in Newborns

**DOI:** 10.3389/fcimb.2020.00332

**Published:** 2020-07-10

**Authors:** Tilman E. Klassert, Cristina Zubiria-Barrera, Stefanie Kankel, Magdalena Stock, Robert Neubert, Fabian Lorenzo-Diaz, Norman Doehring, Dominik Driesch, Doris Fischer, Hortense Slevogt

**Affiliations:** ^1^Host Septomics, ZIK Septomics Research Center, Jena University Hospital, Jena, Germany; ^2^Institute of Human Genetics, Jena University Hospital, Friedrich Schiller University, Jena, Germany; ^3^Genomics and Health Group, Department of Biochemistry, Microbiology, Cell Biology, and Genetics, University of La Laguna, San Cristóbal de La Laguna, Spain; ^4^Abteilung für Geburtshilfe und Gynäkologie, Krankenhaus Sachsenhausen, Frankfurt, Germany; ^5^BioControl Jena GmbH, Jena, Germany; ^6^Zentrum für Kinder- und Jugendmedizin/Schwerpunkt Neonatologie, Universitätsklinikum Frankfurt a.M., Frankfurt, Germany

**Keywords:** newborns, microbiome, antibiotic resistance gene, neonatology, 16S rDNA sequencing

## Abstract

Several studies have recently identified the main factors contributing to the bacterial colonization of newborns and the dynamics of the infant microbiome development. However, most of these studies address large time periods of weeks or months after birth, thereby missing on important aspects of the early microbiome maturation, such as the acquisition of antibiotic resistance determinants during postpartum hospitalization. The pioneer bacterial colonization and the extent of its associated antibiotic resistance gene (ARG) dissemination during this early phase of life are largely unknown. Studies addressing resistant bacteria or ARGs in neonates often focus only on the presence of particular bacteria or genes from a specific group of antibiotics. In the present study, we investigated the gut-, the oral-, and the skin-microbiota of neonates within the first 72 h after birth using 16S rDNA sequencing approaches. In addition, we screened the neonates and their mothers for the presence of 20 different ARGs by directed TaqMan qPCR assays. The taxonomic analysis of the newborn samples revealed an important shift of the microbiota during the first 72 h after birth, showing a clear site-specific colonization pattern in this very early time frame. Moreover, we report a substantial acquisition of ARGs during postpartum hospitalization, with a very high incidence of macrolide resistance determinants and *mecA* detection across different body sites of the newborns. This study highlights the importance of antibiotic resistance determinant dissemination in neonates during hospitalization, and the need to investigate the implication of the mothers and the hospital environment as potential sources of ARGs.

## Introduction

In the last decade, many studies have been conducted to increase the understanding of the early bacterial colonization in newborns. These studies identified several factors of the mother-child-axis to play a crucial role in the microbiome colonization and its maturation in infants, including the mode of delivery (*via naturalis* or through *caesarian* (*c)-section*), breast or formula feeding and antibiotic exposure of the mother during pregnancy or at the time of birth (Dominguez-Bello et al., 2010; Bäckhed et al., 2015; Nuriel-Ohayon et al., 2016; Nogacka et al., 2017). Other aspects of the early microbiome acquisition are controversial in literature, such as the potential sterility of the fetal environment in mammals (Perez-Muñoz et al., 2017). A few studies have challenged the sterile womb dogma, suggesting an intrauterine microbial colonization (Romano-Keeler and Weitkamp, 2014; Stinson et al., 2019). However, it is still unclear if the bacterial colonization of the newborn occurs prior, during and/or after birth (Lim et al., 2018). In any of these scenarios, the maternal microbiome plays the central role in the early microbial colonization of the newborns.

Apart from the maternal microbiome, also the immediate environment at birth has been reported to have an important impact on the early bacterial colonization of neonates (Chong et al., 2018). As a consequence, microbes from the hospital environment (e.g. neonatal intensive care units) have been found to resemble those in the gut of infants and *viceversa* (Brooks et al., 2014). Such microbial interchange between newborns and the immediate environment can lead to colonization and spread of pathogenic bacteria in neonatal intensive care units, often involving multiresistant bacteria (Touati et al., 2009).

The increasing prevalence of antibiotic resistant bacteria has become a major health concern in hospitals in general (Seale and Millar, 2014), and in the perinatal environment in particular (Patel and Saiman, 2010; Ramirez and Cantey, 2019). Several studies report antibiotic resistance genes (ARGs) or resistant bacteria in oral or fecal samples of neonates as early as 1 week after birth (Gosalbes et al., 2016; Gomez-Arango et al., 2017). The identification of risk factors for the acquisition of antibiotic resistant bacteria in newborns has become the focus of many studies in recent years (Moore et al., 2013; Seale and Millar, 2014; Nogacka et al., 2017). Cases of neonatal infections with antibiotic resistant bacteria are steadily increasing and leading to high morbidity and mortality among newborns. The acquisition of these bacteria either during neonatal hospitalization, perinatal vertical transmission and/or antibiotic exposure during gestation or at birth, represent a serious risk for neonatal infections with compromised therapeutic options (Seale and Millar, 2014). Therefore, a clear understanding of how and to which extent bacteria containing ARGs colonize newborns in the first days after birth is essential to minimize risk factors and to control fatal infections.

Most of the studies addressing antibiotic resistance determinants or resistant bacteria in neonates focus on the presence of ARGs from a specific group of antibiotics or on particular pathogens, such as penicillin-resistant *Streptococci*, Group B *Streptococcus* or extended spectrum ß-lactamase bacteria. In this study we aimed to investigate site-specific acquisition of a high variety of antibiotic resistance determinants during the first 3 days after birth. Thus, we analyzed in parallel different body sites from neonates and their mothers for the presence of 20 different ARGs coding for resistances against antibiotic classes with extended use in neonatology or with reported detection of resistance-determinants in older infants (Fanos and Dall'Agnola, 1999; Gosalbes et al., 2016). These antibiotic classes included ß-lactams (cephalosporins and carbapenems), aminoglycosides, macrolides, quinolones, tetracyclines and glycopeptides. In addition, we investigated the dynamics of the early bacterial colonization of neonates during the first 72 h of life.

## Materials and Methods

### Sample Collection

For this study, a total of 108 swab samples from 12 mother-child pairs were collected at a level IV (national) obstetric hospital in Frankfurt a.M. The study was approved by the local institutional ethics committee, and mothers gave written informed consent in accordance with the Declaration of Helsinki. Criteria for inclusion of the mothers were: (i) length of gestation >37 weeks, (ii) non-smokers, (iii) absence of premature blistering, (iv) absence of antibiotic treatment during pregnancy. Eight of the mothers gave birth *via naturalis* while four delivered through *c-section*. Mean age of the mothers was 32.3 years (range 24–40). Women undergoing *c-section* received a single dose (1,000 mg) of prophylactic ampicillin 20 min before intervention. No further perinatal antibiotics were administered to any newborn or naturally birthing mother included in this study. Sample material from the mothers (vagina, pharynx and rectal swabs) was taken before birth. The newborn's oral mucosa (pharynx) and skin (axilla) samples were taken within minutes after delivery and again after 72 h. Rectal swabs of newborns were taken within the first 24 h (after meconium passage) and after 72 h. After sample collection, swabs were immersed in 750 μl lysis solution (Power DNA Isolation kit, MoBio) and stored at −80°C until further analyses.

### DNA Extraction

Genomic DNA was isolated from each swab sample using the MoBio Power DNA Isolation kit according to manufacturer's instructions. Extracted DNA was quantified using a NanoDrop spectrophotometer (Thermo Fisher Scientific) and stored at −20°C for further experiments.

### 16S rDNA Library Preparation and Sequencing

Fecal, vaginal, oral and skin samples were analyzed by high-throughput amplicon sequencing using an Ion Torrent PGM platform (Thermo Fisher Scientific). The variable bacterial V4 region of the 16S rDNA gene was amplified using the F515 and R806 primers as described previously (Caporaso et al., 2012) with modifications to match the requirements of the Ion Torrent library preparation (i.e., Illumina adapter sequences were replaced by Ion Torrent adapter sequences). PCR conditions included an initial denaturation step at 94°C and 35 cycles of denaturation (94°C for 30 s), annealing (50°C for 30 s) and elongation (72°C for 30 s). The whole library construct for the amplification step included the specific primers of the 16S rDNA region, the Ion Adapter A and P1 sequences and a Golay barcode in the reverse primer construct, as follows: forward primer (CCTCTCTATGGGCAGTCGGTGATGTGCCAGCMGCCGCGGTAA) and reverse primer (CCATCTCATCCCTGCGTGTCTCCGACTCAG-NNNNNNNNN-CGGACTACHVGGGTWTCTAAT; where N indicates the barcode sequence location). Barcoded PCR products of ~380 bp were quantified using an electronic gel electrophoresis device (Tape Station 2,200, Agilent) and purified with AMPure XP beads (Beckman Coulter) following manufacturer's instructions. PCR-controls and experimental blank controls were run alongside the mother-child samples during the whole NGS-pipeline to keep track of potential contamination sources. Final libraries were normalized, pooled and loaded on an Ion 318 Chip, and sequenced on the Ion Torrent PGM platform (Thermo Fisher Scientific).

The resulting fastq files were quality checked using FastQC (Andrews, 2010) and trimmed using Trimmomatic (Bolger et al., 2014). The sequences were then analyzed using the QIIME pipeline (Caporaso et al., 2010). Sequences for each sample were demultiplexed and Operational Taxonomic Units (OTUs) were generated by open-reference OTU picking with 97% similarity using the SILVA database (release 132). Summaries of the taxonomic classification were limited to sequences present at relative abundance greater or equal to 0.5% within the samples. A significant impact of contamination sources during the NGS-pipeline could be discarded after comparison of negative controls with newborn samples, assessed by analysis of similarities (ANOSIM). The α-diversity measurements included PD_whole tree and Shannon metrics. Principal coordinate analyses (PCoA) of unweighted UniFrac distances were performed for β-diversity measurements.

### Screening for Antibiotic Resistance Genes

ARGs were selected based on their clinical relevance in neonatology and/or their documented detection during infancy. Thus, different antibiotic classes with extended use in neonates, such as ß-lactams (cephalosporins and carbapenems), aminoglycosides, macrolides, quinolones, and glycopeptides (Fanos and Dall'Agnola, 1999) were covered by the inclusion of the following resistance determinants: *ermA, ermC, mefA, bla*_CTXM−1_, *bla*_CTXM−9_, *bla*_SHV_, *bla*_KPC_, *bla*_VIM−1_, *bla*_NDM_, *bla*_LAT_, *bla*_OXA−48_, *aacC1, aphA6, qnrB-1, vanB, oprM*. In addition, we addressed the expression of tetracycline ARGs (*tetA, tetB*) and the methicillin-resistance determinant *mecA*, as their expression has been documented in gut samples of older infants (Gosalbes et al., 2016). The detection of these 20 ARGs in the maternal and neonate DNA samples was performed by qPCR using specific commercially available TaqMan assays (Microbial DNA qPCR Assays, Qiagen Ref# 330261). The specific assay references and details for each gene are shown in Table S1. The PCR reactions were set up on a CAS-1,200 pipetting robot (Qiagen) and run on a Corbett-Rotor Gene 6,000 device (Qiagen). Thermal PCR conditions included an initial activation step at 95°C for 10 min, and 40 cycles of a denaturation step (95°C for 15 s) and an annealing/extension step (60°C for 2 min). Samples were considered as positive for a specific ARG when the achieved cycle threshold (Ct) was <35.

### Statistical Analysis

For pairwise comparisons between different groups of the dataset, two-tailed Student's *t*-test was performed (threshold for significance *P* < 0.05) using GraphPad Prism 5.0 (GraphPad Software).

## Results

### Microbiome Analysis

**Figure 1:**
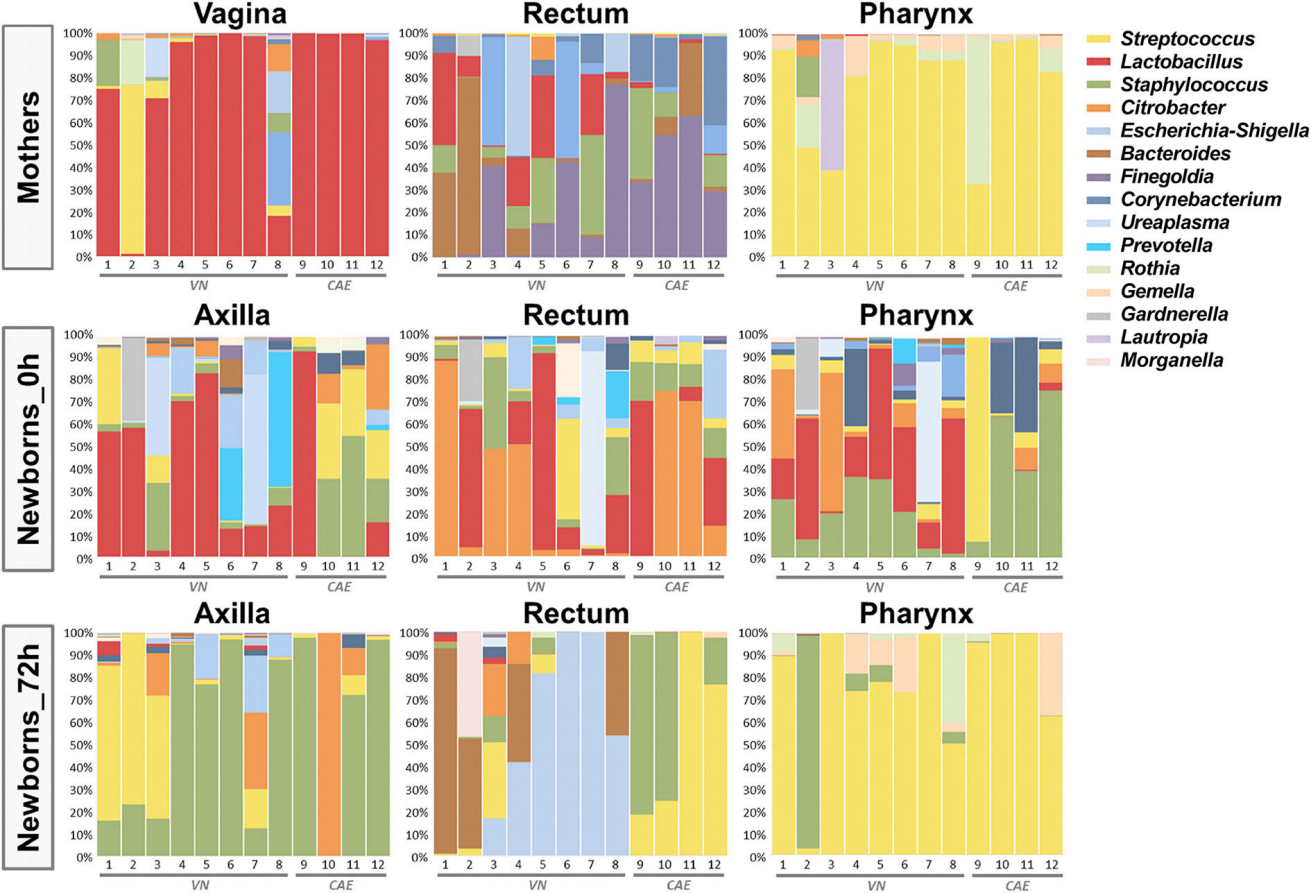
Taxonomic summary depicting the distribution of the microbiome of the 12 mother-child pairs. Bar charts show the relative microbial distribution at genus level for all different analyzed body sites (VN, *via naturalis*; CAE, *cesarean section*).

The microbiome analysis of the 108 samples was performed by targeted sequencing of the V4 region of the 16S rDNA gene using QIIME (Caporaso et al., 2010) for its taxonomic characterization. The results showed a dominance of the five main phyla *Firmicutes, Proteobacteria, Bacteroidetes, Actinobacteria* and *Tenericutes* across all samples. At this taxonomic level, a clear shift in the microbiota distribution of the newborns could already be observed only 3 days after birth, with a slight increase in the relative abundance of *Firmicutes* across all sites (58.7% at birth vs. 71.2% after 72 h), a significant decrease of *Actinobacteria* (7.8% at birth vs. 2.4% after 72 h; *p* = 0.028) and an almost complete loss of *Tenericutes* in this time frame (7.9% at birth vs. 0.3% after 72 h; *p* = 0.045; Supplementary Figure S1). These microbiota changes within the first 72 h of life were further confirmed at lower taxonomic levels as shown in the summary across all samples in Figure 1. The taxonomic analysis also included the samples from each mother (vaginal, stool and pharynx) to account for the bacterial communities which might have an immediate impact on the early colonization of the offsprings. As expected, vaginal samples from the mothers were dominated by *Lactobacillus spp*. (relative abundance of 79.6%), and an important carryover of these bacteria could be detected across all sample sites in infants born *via naturalis* (Figure 1).

When analyzing neonate microbiota by sampling sites immediately after birth, we observed that the *Lactobacillus* carryover was especially dominant in the samples taken from the axillae, which were likely most exposed in the birth canal. In contrast, the rectum samples were dominated by *Citrobacter*, while *Staphylococcus* was the dominant genus in the pharynx samples. For all sampling sites, a significant shift in the microbiota composition was observed after only 72 h, leading to a site-specific colonization pattern. At that time point, the axilla samples were mainly colonized by *Staphylococcus*. The pharynx samples were dominated by *Streptococcus*, thereby resembling the taxonomic distribution of the maternal pharynx samples. Interestingly, the analysis of the rectal samples at this early time point revealed significant differences between the delivery modes. After 72 h, the gut microbiota of neonates born *via naturalis* was dominated by *Bacteroides* and *Escherichia*. In contrast, the *c-section* babies showed a gut microbiota rich in *Streptococcus* and *Staphylococcus* (Figure 1).

**Figure 2:**
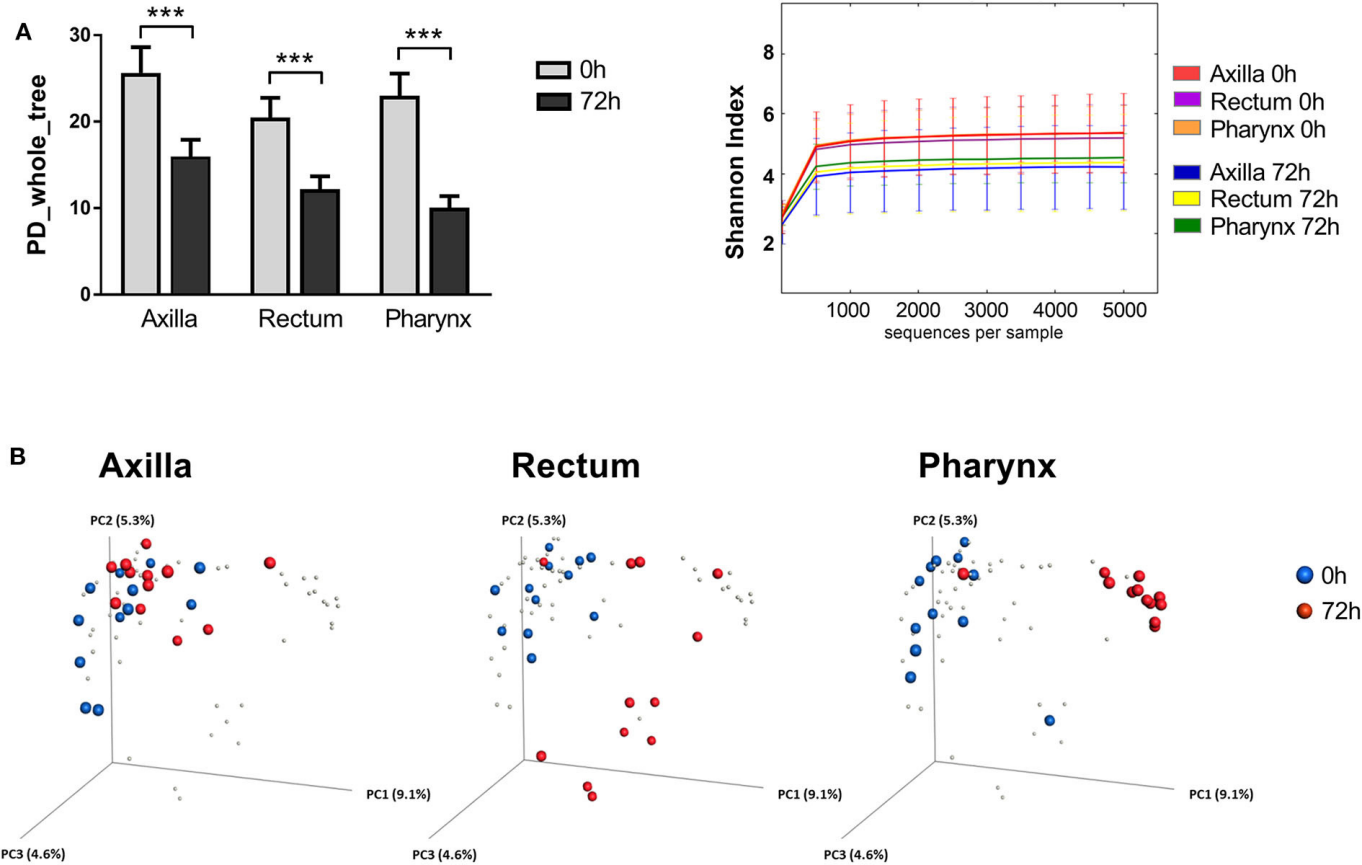
Diversity metrics of the newborn's microbial communities at the time of birth and after 72 h of life. **(A)** Comparison of the α-diversity of the microbial communities from axilla, rectum and pharynx between the two analyzed time points (0 vs. 72 h). Shown are the phylogenetic diversity (left) and the Shannon index (right), respectively (***p < 0.005). **(B)** PCoA analysis of the newborn microbiome. Each point corresponds to a community colored according to the time point of sampling (0 h in blue vs. 72 h in red).

The selective bacterial colonization and differential adaptation to the new site-specific environment of the newborns became apparent also by the reduction of the α-diversity after 72 h. As shown in Figure 2A, the phylogenetic diversity (PD) decreased in a significant manner in all 3 analyzed body sites when comparing the microbial composition at the time of birth and after 72 h (Axilla *p* = 3 × 10^−6^; Rectum *p* = 2 × 10^−6^; Pharynx *p* = 2 × 10^−6^). The diversity also decreased significantly over time when using the Shannon Index as α-diversity metric (Axilla *p* = 1 × 10^−6^; Rectum *p* = 1 × 10^−6^; Pharynx *p* = 1 × 10^−6^; Figure 2A). In a next step, we examined the β-diversity metric in order to statistically measure the distances between the two analyzed time points. The PCoA plots in Figure 2B show a site-specific clustering of the microbiome samples of the newborns after only 72 h of life. Moreover, the microbial community at this time point already differs significantly from its composition at the time of birth in all three body sites (ANOSIM: Axilla *p* = 0.002; Rectum *p* = 0.001; Pharynx *p* = 0.001).

In summary, we could detect significant microbiome changes across different body sites of newborns as early as 72 h after birth, with a site-specific colonization of bacteria from different taxa and a concomitant reduction of the α-diversity.

### Detection of Antibiotic Resistance Genes (ARGs)

**Figure 3:**
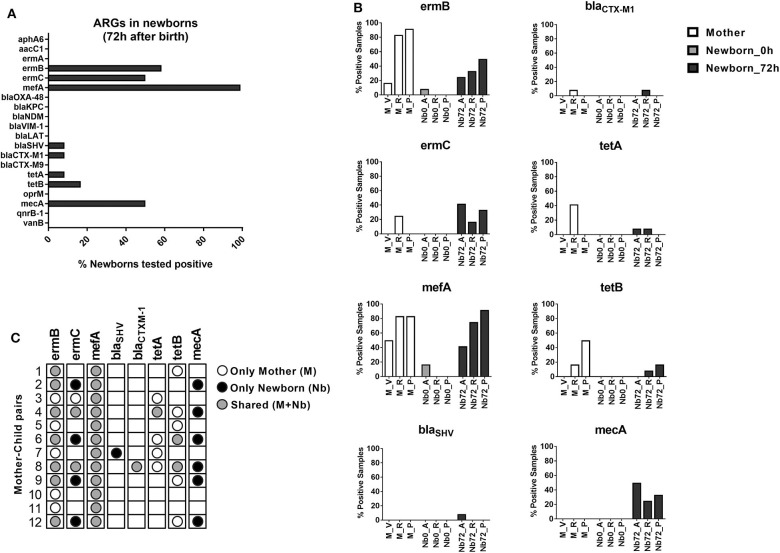
ARG detection in newborns. **(A)** Bar chart depicting the ARG expression in newborns 72 h after birth. Bars represent percentage of newborns with at least one positive sample. **(B)** Detailed view on the distribution of positively tested ARGs across the different sample sites of mothers and newborns at the time of birth and after 72 h (M, mother; Nb0, newborn at time of birth; Nb72, newborn after 72 h; V, vagina; A, axilla; R, rectum; P, pharynx). Bars represent the percentage of positive samples in each case. **(C)** Dot plot showing the presence of ARGs in each mother-child pair, indicating whether the resistance-determinants were detected only in the maternal samples (white circle), only in the newborn samples (black circle) or shared by both (gray circle).

In a next step, we aimed to investigate whether and to which extent these early microbiome changes, which occur in a clinical environment, might be associated with the acquisition of antibiotic resistance determinants. Therefore, all mother-child pairs were tested for the presence of twenty different ARGs using specific real-time qPCR assays (see Table S1 in the Supplementary Material). While 12 of the twenty ARGs were not detected in any of the newborn's samples, eight ARGs were tested positive in at least one the newborn's samples 72 h after birth (Figure 3A). The ARGs found to be present in newborns included the genes coding for ribosomal RNA methyltransferases (*ermB* and *ermC*), a ribosomal protection protein (*mefA*), β-lactamases (*bla*_SHV_ and *bla*_CTXM−1_), tetracycline efflux pumps (*tetA* and *tetB*) and the penicillin-binding protein PBP2a (*mecA*).

Genes conferring resistance to macrolides showed the highest dissemination in our sample set, with 100% of the newborns analyzed being positive for *mefA*, 58% for *ermB*, and 50% for *ermC*. The β-lactamase coding genes *bla*_SHV_ and *bla*_CTXM−1_ were found in only 8.3% of the newborns each, while the tetracycline-resistance genes *tetA* and *tetB* showed an incidence of 8.3 and 16.7%, respectively. Surprisingly, the MRSA-associated gene *mecA*, showed a very high presence in our population, with at least one positive sample in 50% of the newborns analyzed after 72 h (Figure 3A).

When analyzing the presence of resistance determinants at the different time points (0 vs. 72 h), our results show a very low incidence of ARGs immediately after birth. In contrast, a high presence of ARGs across different body sites was observed for the samples taken after 72 h (Figure 3B). These results suggest that the acquisition of ARGs is concomitant with the early bacterial colonization and site-specific adaptation processes that occurs within the first 3 days of life. Comparison of positive ARG counts at the different body sites showed highest presence of ARGs in the pharynx (27 total ARG counts across all newborn's pharynx samples). However, the highest ARG-diversity in our population, as measured by the number of different ARGs detected, was found in the rectum samples (seven different ARGs; see Supplementary Figure S2).

One major source for the bacterial colonization of newborns is the maternal microbiota. In order to explore the likelihood of a vertical transmission of ARGs, the samples from the mother (vagina, rectum and pharynx) were also included in the ARG detection analyses. Positive signals in these samples were found only for six out of the 20 tested ARGs (*ermB, ermC, mefA, bla*_CTXM−1_, *tetA*, and *tetB*, see Figure 3B), representing a high overlap with the genes found in the newborn samples. In order to evaluate the likelihood of a direct transmission from the mothers to their offsprings, we then analyzed each mother-child pair independently. As shown in Figure 3C, the macrolide-resistance determinants *ermB* and *mefA* were detected in all 12 mother-child pairs. For *ermB*, 7 of the 12 pairs showed a shared presence (mother and child) of the gene. For *mefA* the likelihood of vertical transmission was even higher, with all 12 pairs displaying a shared presence of the gene in mother and neonate samples. The *bla*_CTXM−1_ gene was only detected in one mother-child pair, where it was shared by both. Other ARGs showed lower rates of shared expression among mother-child pairs. Only two of the positive *ermC* pairs showed a shared presence in mother and child samples, while in four pairs the gene was detected only in the newborn. Moreover, the resistance-determinants *bla*_SHV_ and *mecA* were detected only in newborn samples. On the other hand, the tetracycline-resistance genes *tetA* and *tetB* showed a high presence in maternal samples, but a low rate of transmission to their offsprings (Figure 3C). These results suggest a distinct likelihood for the transferability of certain gene groups between mothers and their neonates, which might be dependent on their presence in the early colonizing taxa described in our microbiome analysis. However, these results might also point toward an additional environmental source for the acquisition of particular resistance determinants, such as *mecA* and *bla*_SHV_, during the early development of the newborn microbiota.

## Discussion

Several aspects influence the early colonization dynamics of the infant microbiome. Different studies have already highlighted the importance of factors such as the hormonal or metabolic changes during pregnancy, the antibiotic exposure, the mode of delivery or the infant feeding patterns in the microbial colonization of newborns (Dominguez-Bello et al., 2010; Bäckhed et al., 2015; Nuriel-Ohayon et al., 2016; Nogacka et al., 2017). Also the hospital environment might shape the microbiome during the first hours after birth (Chong et al., 2018). Although the maturation process of the infant microbiome is dynamic and lasts for several years, the pioneer microbiota of the neonate already plays a crucial role and has a persistent impact on the microbiome development (Gasparrini et al., 2019). Due to the slow maturation kinetics of the infant microbiota, most studies addressing its developmental process have focused on larger periods of weeks or months after birth (Chu et al., 2017; Moore and Townsend, 2019). However, it seems logical to think that the first hours after birth represent the primary exposition to major sources of microorganisms and might have a critical impact on the early microbial colonization of newborns. In the present study, we characterized the neonate microbiome within the first 72 h after birth, when the hospital environment might play an additional role not only in the microbial colonization but also in the potential acquisition of the first ARGs. Moreover, this early microbiome analyses were carried out in three different body sites targeting the gut-, the oral-, and the skin-microbiome of the newborns, while analyzing the presence of 20 different ARGs. To our knowledge, this is the first study to address such an amount of ARGs across different body sites in newborns.

The 16S rDNA sequence analysis of the different neonate samples revealed an important shift of the microbiota during the first 3 days of life. The bacterial colonization during this early time frame was highly site-specific. After 72 h, *Staphylococcus* species dominated the axilla samples, while *Streptococcus* was the most prominent taxa in almost all pharynx samples, regardless delivery mode. In contrast, the rectum samples showed a distinct microbiota pattern between *via naturalis* and *c-section* delivery. The gut colonization of *cesarean*-born neonates was characterized by *Firmicutes* genera such as *Staphylococcus* and *Streptococcus*. On the other hand, the naturally born babies showed increased presence of typical gut taxa, such as *Bacteroides* and *Escherichia*. This is in agreement with previous studies reporting a faster establishment/development of the characteristic gut microbiota in naturally born infants (Shao et al., 2019). It has been proposed that the perturbation observed in the gut colonization of *c-section* babies might be a consequence of the disrupted transmission of maternal *Bacteroides* strains, and the concomitant colonization by different opportunistic bacteria from the environment (Shao et al., 2019). However, the colonization pattern observed in the *c-section* neonates might also derive, at least in part, from the single dose prophylactic antibiotic administered to the mothers. Recent evidence showed that intrapartum antimicrobial prophylaxis might lead to altered intestinal microbiota establishment in the neonates during the first weeks of life, with an increased Firmicutes/Bacteroidetes ratio (Nogacka et al., 2017). It is likely that both aspects, delivery mode and antibiotic prophylaxis, contribute to the colonization differences observed between groups.

The microbiota shift in the first days after birth was characterized by a significant loss of diversity as shown by different α-diversity measures. Various studies have documented a steady increase of microbial diversity during the first months of microbiome development in infants (Bokulich et al., 2016; Gasparrini et al., 2019). However, as stated before, these studies investigated rather large time periods of weeks/months after birth. The observed decrease of microbial diversity in our study is likely a consequence of the initial adaptation of the different taxa to each niche and the selective pressure of the site-specific environment on the newly establishing flora. Once this selective process has concluded, the gain of novel bacteria and the community dynamics might steadily lead to an increase in the microbiota diversity over the following months and years of life as previously reported (Gasparrini et al., 2019).

In a next step, we investigated whether ARGs conferring resistance to different antibiotics were present at the different analyzed body sites at such an early stage of the microbiome development. In total, we were able to detect eight of the 20 tested ARGs in any of the newborn samples. While very low presence of ARGs was detected immediately after birth, an important dissemination of different ARGs was detected after only 72 h (Figure 2B). The elevated incidence of ARGs in newborns found in this study is in line with the results obtained recently by Gasparrini et al. (2019), when analyzing stool samples of infants during their first months of life. Using functional metagenomics approaches, the authors detected the presence of resistance determinants in the gut of infants against all of the tested antibiotic groups, including quinolone, polymixin, aminoglycoside, β-lactam, tetracycline and amphenicol. Moreover, the antibiotic determinants were detected in stool libraries from both preterm antibiotic-exposed infants and near-term antibiotic-naive infants (Gasparrini et al., 2019). Our results now show that the acquisition of such amounts of ARGs seems to start taking place during the first hours of life. Moreover, we add on the findings by Gasparrini et al. (2019), reporting a high presence of macrolide resistance ARGs. This group of determinants showed the highest incidence in our samples, with *mefA* being detected in all of the tested newborns 72 h after birth. A few studies have associated this gene to several *Streptococcus* species (Bley et al., 2011; Hraoui et al., 2012). The high incidence of *mefA* might therefore be related to the enrichment of *Streptococcus spp*. observed in the 16S rDNA profiles of the neonate samples collected after 72 h, especially in the pharynx, where the presence of *mefA* was most prominent. Macrolides are used as first line drugs in infants to treat infections such as trachoma, pertussis and *Campylobacter* enteritis (Tiwari et al., 2005; Amza et al., 2017; Same and Tamma, 2018). Accumulation of macrolide resistance determinants in the microbiome of newborns might therefore have clinical implications later in life.

The mother's microbiome is thought to be the major source of ARGs for the establishing microbiome of the neonates. In our study, samples from different body sites of the mothers were included in the analyses in order to track potential ARG transmission ways by comparing paired mother-newborn samples. The macrolide-resistance determinants *ermB* and *mefA* were found to be shared among mother and offspring samples in most of the analyzed mother-child pairs. These results suggest a high likelihood of vertical transmission of these genes. On the other hand, genes such as *bla*_SHV_ and *mecA* were found to be present only in newborn samples. The latter one (*mecA*) showed a very high incidence, with 50% of the newborns being tested positive in at least one body site after 72 h. This gene is widely disseminated in *Staphylococcus aureus*, and confers resistance to methicillin (Wielders et al., 2002). This methicillin-resistant *S. aureus* (MRSA) is one of the most important causes of hospital infections worldwide (Diekema et al., 2001), with increasing relevance also in the perinatal environment (Shirai et al., 2017; Washam et al., 2018). It might be hypothesized, that the genes *bla*_SHV_ and/or *mecA*, which were not detected in the mother samples, could have been acquired from the hospital environment. Its potential as exogenous source has already been reported for particular ARGs or resistant strains (Touati et al., 2009; Brooks et al., 2014). Two of the main limitations of this study include the lack of environmental samples and the inability to assign detected ARGs to specific bacteria. The hypothesis of hospital acquired ARGs should therefore be validated in further studies including environmental samples. Culture-based methods or shotgun metagenomics might thereby help to identify the specific microorganisms carrying the antibiotic resistance determinants.

In conclusion, this study describes the early microbiome dynamics in different body sites of newborns during the first 72 h after birth. In addition, we report an important acquisition of ARGs during this short time period, with a very high incidence of macrolide resistance determinants and *mecA* expression across the tested body sites. Further multicenter studies should validate our findings and investigate the potential implication of the hospital environment on the reported acquisition of ARGs by neonates during the first hours of life.

## Data Availability Statement

The datasets generated for this study can be found in the SRA database under the accession number: PRJNA615809 [https://www.ncbi.nlm.nih.gov/sra/PRJNA615809].

## Ethics Statement

The studies involving human participants were reviewed and approved by the Medicine Department of the University Hospital Frankfurt a.M. Written informed consent to participate in this study was provided by the participants' legal guardian/next of kin.

## Author Contributions

TK, DF, and HS conceived and designed the study and experiments. TK and CZ-B wrote the manuscript. TK, CZ-B, SK, and RN conducted the experiments. TK, CZ-B, SK, MS, FL-D, and DD analyzed the data. ND and DF recruited the study cohort and collected samples. All authors reviewed and edited the manuscript. All authors contributed to the article and approved the submitted version.

## Conflict of Interest

DD was employed by BioControl Jena GmbH. The remaining authors declare that the research was conducted in the absence of any commercial or financial relationships that could be construed as a potential conflict of interest.
