# Supplementary material for: Early Bacterial Colonization and Antibiotic Resistance Gene Acquisition in Newborns
Source: Front Cell Infect Microbiol. 2020 Jul 10;10:332. doi: 10.3389/fcimb.2020.00332 (PMC7366792; doi:10.3389/fcimb.2020.00332)
Supplement: Supplementary file 1 [file Table_1.DOCX]

Supplementary Material

# Supplementary Table

**Table S1: Overview of the 20 antibiotic resistance genes**

| **Gene** | **Antibiotic classification/Gene description** | **Detection of ARGs** | **REFERENCE**  **(Qiagen Assay ref #)** |
| --- | --- | --- | --- |
| tetA | Tetracycline |  | BPAR00449A |
| tetB | efflux pump |  | BPAR00450A |
| ermA | Macrolide |  | BPAR00442A |
| ermB | Lincosamide |  | BPAR00443A |
| ermC | Streptogramin_b |  | BPAR00444A |
| mefA |  |  | BPAR00445A |
| mecA | Beta-lactam resistance |  | BPAR00374A |
| blaCTX-M-1 Group | Class A beta-lactamase | Detects CTX-M-1 type (37 variants) | BPAR00377A |
| blaCTX-M-9 Group |  | Detects CTX-M-9 type (40 variants) | BPAR00379A |
| blaSHV (238G240E) |  |  | BPAR00390A |
| blaKPC |  | KPC-1, KPC-2, KPC-3, KPC-4, KPC-5, KPC-6, KPC-7, KPC-8, KPC-9, KPC-10, KPC-11 | BPAR00382A |
| blaVIM-1 Group | Class B beta-lactamase | VIM-1, VIM-2, VIM -3, VIM -4, VIM -5, VIM -6, VIM -8, VIM -9, VIM -10, VIM -11, VIM -12, VIM -14, VIM -15, VIM -16, VIM -17, VIM -18, VIM -19, VIM -20, VIM -23, VIM -24, VIM -25, VIM -26 | BPAR00403A |
| blaNDM |  |  | BPAR00402A |
| blaLAT | Class C beta-lactamase | LAT-1, LAT-2, VIM -3, VIM -4 | BPAR00414A |
| blaOXA-48 Group | Class D beta-lactamase |  | BPAR00423A |
| aacC1 | Aminoglycoside resistance |  | BPAR00367A |
| aphA6 |  |  | BPAR00373A |
| QnrB-1 Group | Fluoroquinolone resistance |  | BPAR00434A |
| VanB | Vancomycin resistance |  | BPAR00451A |
| OprM | Multidrug resistance efflux pump | QnrB1, QnrB 2, QnrB3, QnrB6, QnrB7, QnrB9, QnrB13, QnrB14, QnrB15, QnrB16, QnrB17, QnrB18, QnrB20, QnrB23, QnrB24, QnrB29, QnrB30 | BPAR00447A |

## Supplementary Figures


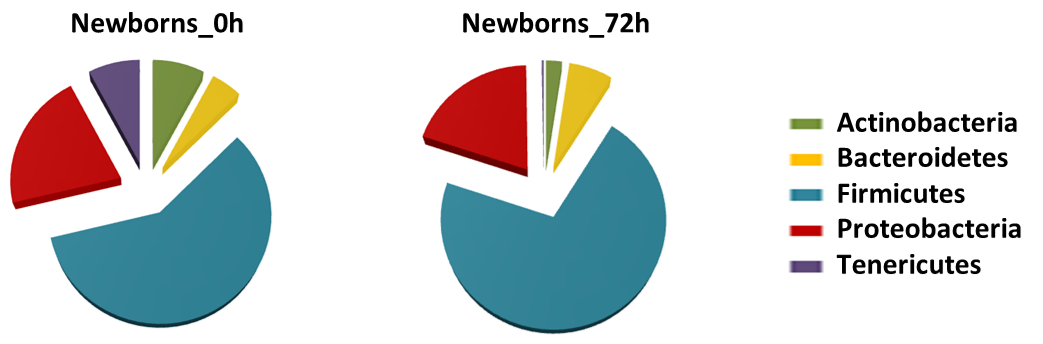


**Suppl. Figure 1. Taxonomic summary of the neonate samples at phylum level.** Relative microbiota distribution of the samples collected immediately (0h) and 72h after birth.


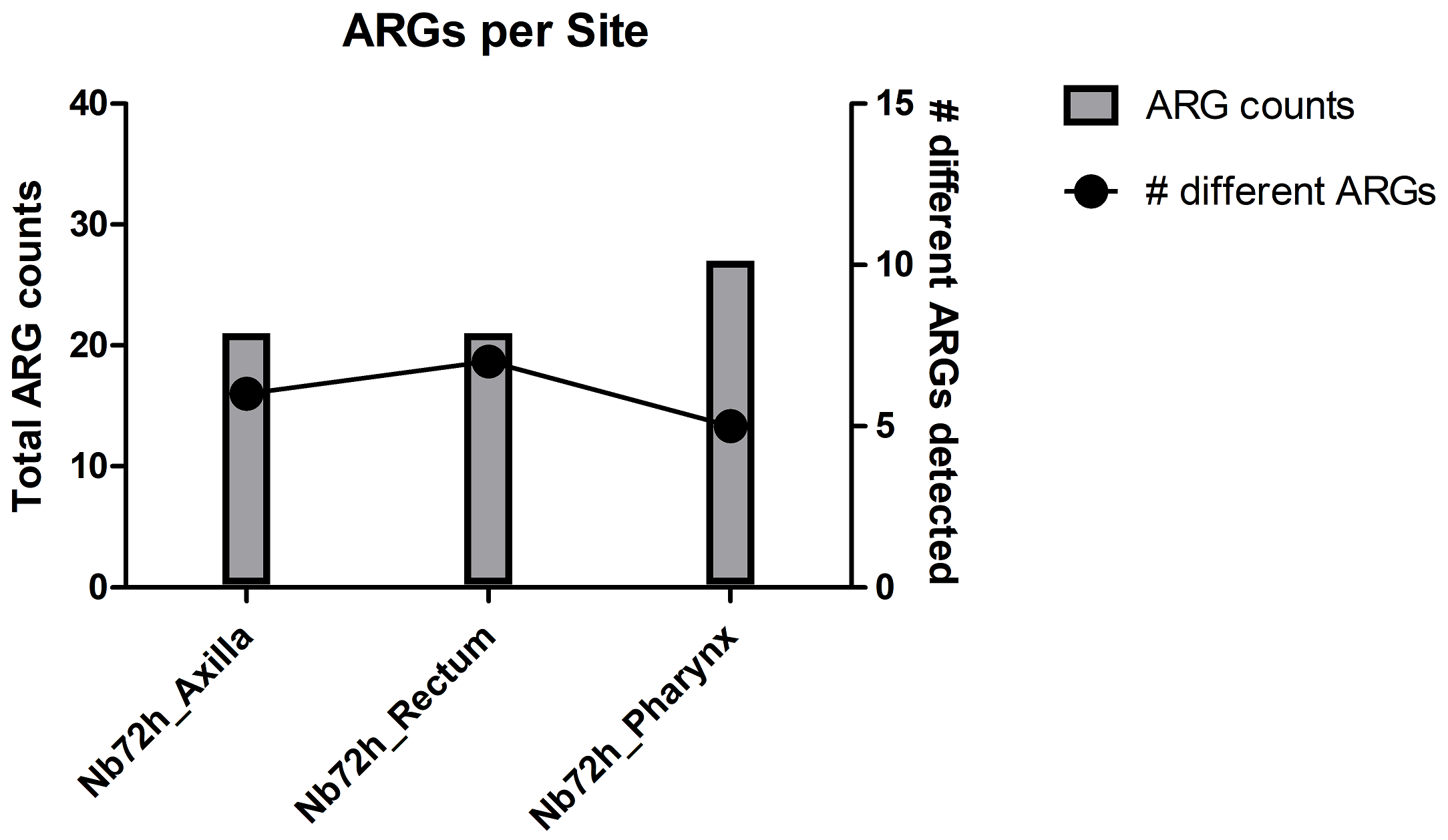


**Suppl. Figure 2. Incidence of ARGs per body site.** Bars represent the total count of ARGs detected in the different body sites of the newborns after 72h (sum of all positive ARG hits across all samples). Dots/lines depict the cumulative amount of different ARGs identified at each body site.
